# Disseminated Intravascular Coagulation After Embolization to Treat Acutely Bleeding Bilateral Massive Angiomyolipoma: A Case Report

**DOI:** 10.1089/cren.2018.0020

**Published:** 2018-07-01

**Authors:** David Carter, Bill Papps, Nicholas R. Brook

**Affiliations:** Department of Urology, The Royal Adelaide Hospital, Adelaide, Australia.

**Keywords:** disseminated intravascular coagulation, renal angiomyolipoma embolization

## Abstract

***Background:*** Hemorrhage from an angiomyolipoma (AML) of the kidney can be life threatening and arterial embolization is the primary treatment. Embolization is less invasive than surgery, is well tolerated, and major complications are rare. We describe a case of disseminated intravascular coagulation (DIC) after embolization of a bleeding renal AML in a 44-year-old man with massive bilateral AMLs. This report aims to highlight the possibility that acute DIC could be a major complication of embolization itself and so should be considered and screened for because, if present, it requires early and aggressive management.

***Case Presentation:*** A 44-year-old man with a history of large bilateral renal AMLs associated with tuberous sclerosis complex presented with visible hematuria and abdominal pain. Renal CT revealed bleeding from the right kidney. Embolization with polyvinyl alcohol and lipiodol was urgently performed. The following day he required multiple blood transfusions and repeat embolization, this time with gelfoam and “tornado” coils. He suddenly developed DIC, cardiovascular collapse and acute renal failure requiring many days in the intensive care unit for inotropic support and renal replacement therapy.

***Conclusion:*** Arterial embolization may be associated with increased risk of DIC in the setting of treating large bleeding renal AMLs. DIC may be a direct or indirect complication of this. The clinician must act quickly to identify this and treat this complication aggressively.

## Introduction and Background

Angiomyolipomas (AMLs) are the most common benign tumors of the kidney. Renal AMLs are choristomas (benign tumors composed of tissues not normally found in the organ). They are composed of dysmorphic blood vessels, muscle cells, and fat cells. About 80% of renal AMLs are sporadic and not associated with any genetic syndrome while ∼10% of people with renal AML have the genetic disease tuberous sclerosis complex (TSC).^1^ TSC affects cellular differentiation, proliferation, and migration early in development, resulting in a variety of hamartomatous lesions that affect many organ systems.

The first-line treatment for acutely hemorrhaging renal AMLs is selective angioembolization. The well-documented complications of this form of treatment include retroperitoneal hemorrhage, dilatation of nearby blood vessels, aneurysm rupture, and need for re-embolization.

We report a case of disseminated intravascular coagulation (DIC) with cardiovascular collapse (CVC) as an immediate major complication of renal AML embolization. In our literature review we did not find any other case of DIC after renal AML embolization.

## Case Presentation

**FIG. 1.**
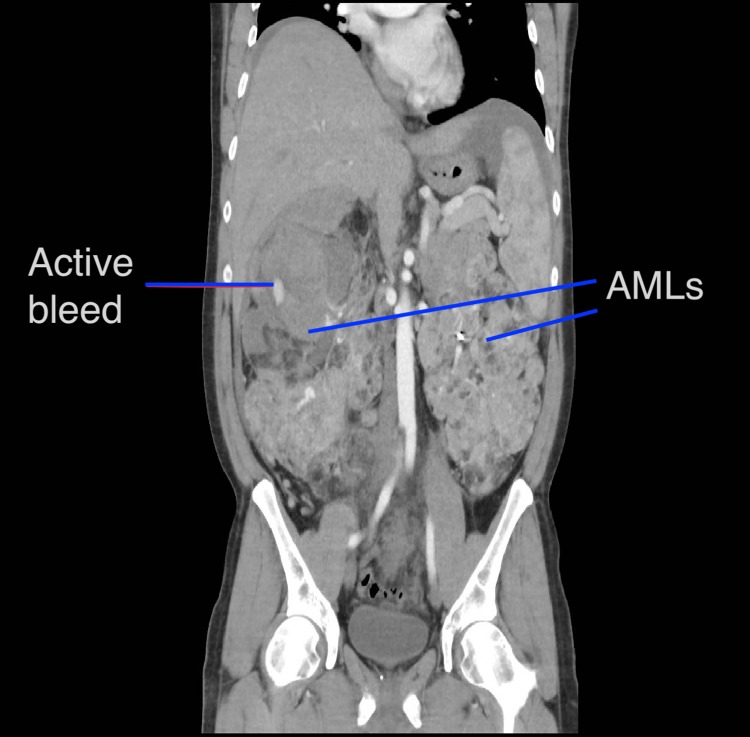
CT showing massive bilateral AMLs and hematoma formation on right upper pole. AML, angiomyolipoma.

A 44-year-old man presented with visible hematuria, right-sided abdominal pain and a distended abdomen on a background of known large bilateral renal AMLs (right 23 × 21 cm, left 21 × 15 cm) (Fig. 1) associated with tuberous sclerosis and a history of multiple previous embolizations, performed without complication.

He had multiple comorbidities including hypertension, a ventriculoperitoneal shunt, and end ileostomy after panproctocolectomy and neoadjuvant radiotherapy in March 2015 for a rectal adenocarcinoma.

His renal function on admission was normal and he was hemodynamically stable initially. Dimercaptosuccinic acid scan was performed, which showed the right kidney contributed 57% and left kidney 43% of the differential renal function, respectively.

**FIG. 2.**
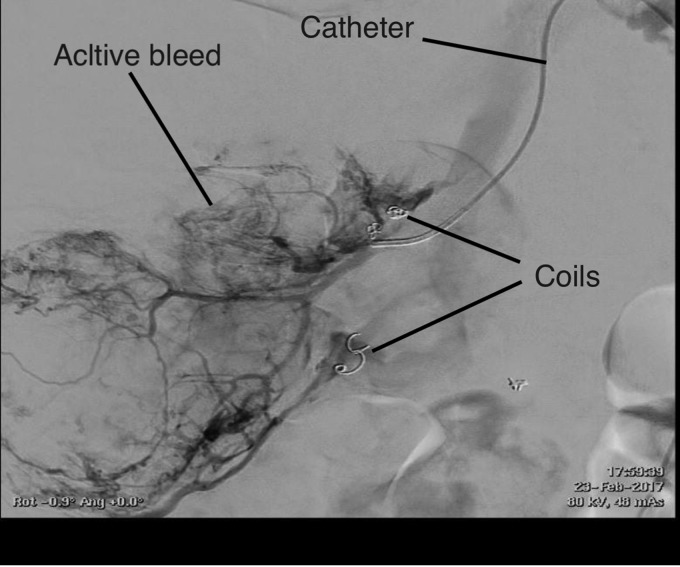
Angiogram, prelipiodol injection. Coils from previous embolizations shown.

Renal CT angiography suggested his right kidney was actively bleeding in the upper pole (Fig. 1) and confirmed on direct angiography (Fig. 2); so decision was made to proceed to selective embolization. Five milliliters of polyvinyl alcohol and 5 ml of lipiodol were used. His hemoglobin fell from 94 to 70 g/L and he was transfused with 2 U of red blood cells. His hemoglobin increased to 84 g/L.

**FIG. 3.**
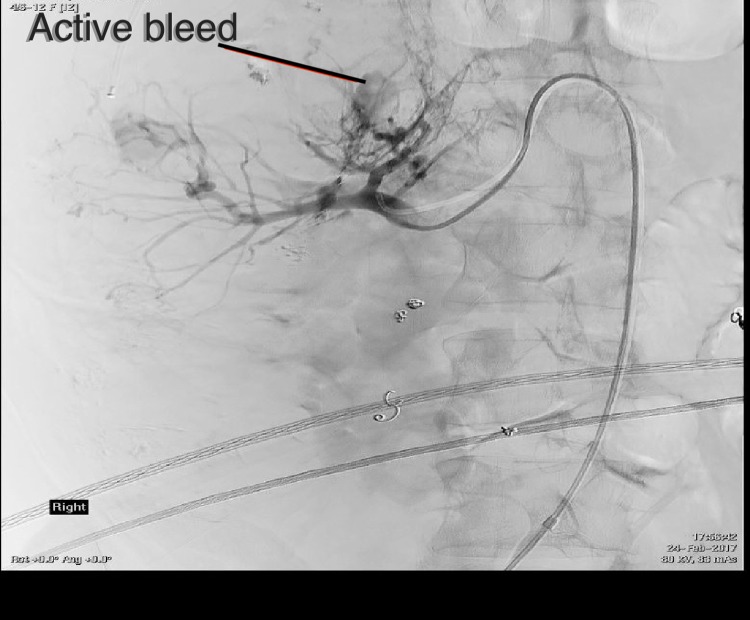
Angiogram, pre-gelfoam, and tornado coils. Bleeding in upper pole identified.

**FIG. 4.**
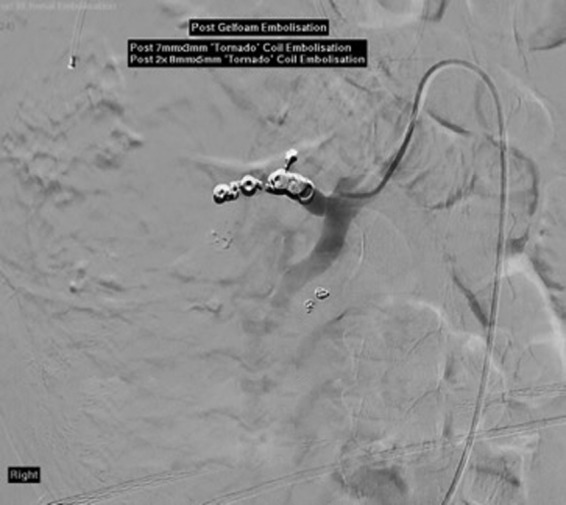
Postembolization. Note absence of contrast extravasation beyond the coils.

He deteriorated the next day, complaining of lethargy, malaise, hematuria, and ongoing abdominal pain. He was febrile, and blood tests revealed a significant fall in hemoglobin level and estimated glomerular filtration rate (eGFR) to 76 g/L and 55 mL/min, respectively. He was then given a dose of gentamicin, 4 U of red blood cells and 2 U of platelets. Repeat CT was nondiagnostic owing to the presence of contrast in his kidneys from the previous intervention. During repeat direct angiography the source of bleeding could be lateralized to the upper pole of the right kidney again. Angiography was also done on the left to ascertain no further bleeding sites. As such it was embolized again; on this occasion with gelfoam and four “tornado” coils (Figs. 3 and 4). He was then transferred to the high dependency unit for closer monitoring as he became hemodynamically unstable.

DIC was discovered postprocedure on coagulation studies with an international normalized ratio (INR) of 1.6, decreased fibrinogen, elevated partial thromboplastin time/prothrombin time, and platelets 62; so was transfused 2 U of fresh frozen plasma and 2 more units of red blood cells. He was oliguric and hypertensive with a rapidly declining eGFR and a four-liter positive fluid balance. He was still febrile so piptazobactam and vancomycin were commenced and was upgraded to intensive care unit care for continuous veno-venous hemodiafiltration (CVVHDF). A three-lumen central venous catheter was inserted into the left femoral vein. CVVHDF commenced that evening and continued for 5 days.

Hemoglobin continued to fall so he was given a further 2 U of red blood cells and 2 U of fresh frozen plasma. He had persistent fevers and hematuria. He was on a fentanyl infusion for abdominal pain. INR rose to 1.8.

A hemolysis screen was performed to account for his ongoing hemoglobin decline; however, this was negative.

After 10 days of supportive care, he began improving clinically and after another 2 weeks was discharged to home. He has been referred to the renal transplant service for consideration of a bilateral nephrectomy and transplant but thus far has remained stable and has not reattended our institution.

## Discussion and Literature Review

AML is a benign mesenchymal tumor of the kidney. The tumor is characterized by aberrant proliferation of dysmorphic blood vessels, smooth muscle, and fat. Ten percent are associated with inheritance of the TSC1 and TSC2 genes, the remainder are sporadic mutations.

Sixty to eighty percent of people with TSC have renal AMLs.

AML tumors have a potential for bleeding complications with increasing size and are usually treated when there is acute hemorrhage, increasing pain or size over 4 cm.^2^

Treatment options for a bleeding renal AML include nephrectomy, embolization, cryoablation, radiofrequency ablation, and enucleation. The primary aim of treatment is achieving hemostasis while causing the least damage to the kidney and thus preserving renal function. Embolization has produced robust results. Polyvinyl alcohol as single agent and ethanol combinations has been documented. Ethanol and polyvinyl alcohol mixture for renal AML embolization has been documented to show sustained mid-term effects.^1^

The difficulties with this case were that that the patient had severe bleeding of a very large AML, and he had multiple previous renal embolizations. It can be seen in the CT images that the renal architecture was very distorted. The second CT was not able to accurately identify the exact location of the bleeding; in this situation we relied on the direct angiographic images at the time of embolization, where both kidneys were checked.

This case demonstrated an episode of DIC and hemodynamic collapse after renal embolization with instillation of polyvinyl alcohol and lipiodol via interventional radiological techniques for a hemorrhaging renal AML. In DIC the coagulation cascade is activated when blood is exposed to tissue factor because of vascular damage and cytokine released from the retained embolized tissue. DIC is expressed as clotting dysfunction with bleeding and concomitant microvascular occlusion. Etiological causes of DIC include trauma, retained ischemic tissue such as pregnancy complications, malignancy, infection, toxic or autoimmune insults, and organ damage.

This patient's DIC may have been caused by the necrotic tissue that remains *in situ* after embolization, similar to how it happens in obstetric complications, notably in placental abruption and miscarriage where the dead fetus remains intrauterine, known as retained dead fetus syndrome.

There are no reports of DIC and CVC after ethanol embolization in renal AML. There are not even any other cases of DIC in patients with renal AML.

DIC and CVC have been previously documented twice with embolization using ethanol for different indications; a case involving embolization with ethanol of an arteriovenous malformation of the lip noted DIC and CVC with failed resuscitation.^3^ More recently in March 2018 a case of DIC after ethanol embolization of an abdominal wall arteriovenous malformation was reported.

There are also many cases in the literature of DIC after uterine artery embolization using various agents to treat postpartum hemorrhage.

Fatal complications after embolization with smaller particles including tris-acryl gelatin have also been documented.^4^ However, no study has shown one agent to be statistically more likely to cause DIC.

Arterial embolization is used as management of renal AMLs and demonstrates low rates of mortality and complications. Despite this, rare complications such as the one demonstrated in this case highlight the potential rapid clinical decline and compromise relating to the possible DIC trigger and resulting CVC.

## Conclusion

DIC is an unusual but life-threatening complication of a hemorrhaging large renal AML. This has not previously been reported. This may be secondary to embolization itself, either from the agents used or from the resulting necrotic tissue. We acknowledge it is possible that DIC was secondary to the large amount of blood loss and subsequent blood transfusion. Vigilance for the signs of DIC is recommended in arterial embolization for large AML. If present, aggressive and early management is required.

## Abbreviations Used

AML: angiomyolipoma
CT: computed tomography
CVC: cardiovascular collapse
CVVHDF: continuous veno-venous hemodiafiltration
DIC: disseminated intravascular coagulation
eGFR: estimated glomerular filtration rate
INR: international normalized ratio
TSC: tuberous sclerosis complex
